# Environmental footprints of food consumption: Protocol for a systematic literature review

**DOI:** 10.1371/journal.pone.0277227

**Published:** 2022-11-07

**Authors:** Camila Valdejane Silva de Souza, Larissa Mont’Alverne Jucá Seabra, Maria Hatjiathanassiadou, Diogo Vale, Gidyenne Christine Bandeira Silva de Medeiros, Dirce Maria Lobo Marchioni, Severina Carla Vieira Cunha Lima, Clélia de Oliveira Lyra

**Affiliations:** 1 Postgraduate Program in Collective Health, Federal University of Rio Grande do Norte, Natal, Rio Grande do Norte, Brazil; 2 Department of Nutrition, Federal University of Rio Grande do Norte, Natal, Rio Grande do Norte, Brazil; 3 Postgraduate Program in Nutrition, Federal University of Rio Grande do Norte, Natal, Rio Grande do Norte, Brazil; 4 Federal Institute of Education, Science and Technology of Rio Grande do Norte, Natal, Rio Grande do Norte, Brazil; 5 Systematic Review and Meta-Analysis Laboratory (Lab-Sys), National Council for Scientific and Technological Development (CNPq), Federal University of Rio Grande do Norte, Natal, Rio Grande do Norte, Brazil; 6 Department of Nutrition, School of Public Health, University of São Paulo, São Paulo, Brazil; Shenzhen University, CHINA

## Abstract

Environmental footprints are indicators that can be used to estimate the impacts of diet on the environment. Since contemporary dietary practices are related to negative environmental impacts, this paper aims to describe a systematic review protocol to investigate the environmental footprints of food consumption by adults and elderly individuals worldwide. This protocol was developed based on the Preferred Reporting Items for Systematic Reviews and Meta-Analyses (PRISMA). Search strategies and records of evidence searched in previously defined electronic databases will be defined. Original, population-based articles investigating the environmental footprints of food consumption by adults and the elderly will be included. Two independent reviewers will conduct the study selection and data extraction steps. Critical appraisal of the included studies will be based on the Newcastle-Ottawa Scale. For data synthesis, a narrative synthesis and, if possible, also a meta-analysis will be performed. The systematic review produced from this protocol will provide evidence for data synthesis of the environmental impact through environmental footprints of food consumption of the adult and elderly population from different territories and the footprint assessment tools used around the world. Therefore, it is a gap that needs to be filled because knowing these impacts will be important to inform the development of public policies that encourage healthy and sustainable food in the face of climate and epidemiological changes.

**PROSPERO registration number**: CRD42021281488.

## 1. Introduction

Modern society’s food choices have been associated with impacts on the environment and the health of individuals [1], since unhealthy eating patterns represent the greatest risk factor for morbidity and mortality worldwide [2]. As a result of globalization, an increase in the consumption of ultra-processed foods and meats has been observed, being associated with the development of some noncommunicable diseases (NCDs) and malnutrition, as well as increased emissions of greenhouse gases (GHG), water pollution, soil degradation and other negative environmental impacts [3–6].

Considering this and other unsustainable patterns of today’s society, the United Nations (UN) has published 17 Sustainable Development Goals (SDGs) to be achieved by nations by 2030, aiming at promoting sustainable actions in order to reduce environmental degradation and unbridled use of natural resources, associated with current life, production and consumption patterns [7].

The development and application of indicators capable of measuring the potential environmental impacts caused by production processes and the passage of man on Earth has been used as tools to identify unsustainable consumption practices. The results obtained through this measurement allow the identification of strategies to reduce the consumption of natural resources [8–10].

Estimating environmental footprints has been an important indicator used in this context, since they aim to measure the traces of environmental degradation caused by humans. Carbon, water, and ecological footprints, among others, allow estimating aspects related to the emission of GHG, water and land use, as well as the extent to which human activities rely upon the planet’s regenerative capacity [11].

Efforts to minimize such environmental damages have stimulated the development of scientific research on environmental impacts related to eating patterns. Studies have shown that healthy diets, based on plant-based foods and lower consumption of meat, especially red meat, have lower environmental footprints [9, 12].

Several studies are identified in the literature that have estimated this damage from environmental footprints. However, it should be noted that different methodologies are used, which sometimes do not allow the assessment of these footprints of individual food consumption. This makes it difficult to understand the relationships between individual diet, environmental impacts, and the health-disease process. Therefore, knowing the environmental impact resulting from populations’ food consumption can support the promotion of healthy and sustainable dietary practices.

Thus, this paper describes the protocol of a systematic review that aimed to investigate the environmental footprints of food consumption in population-based research with adults and older people around the world. The systematic review produced from this protocol will provide a synthesis of the environmental impact data of food consumption of populations in this age group in different territories and of the food footprint assessment tools used worldwide, recognizing the challenges and potentials in using these indicators.

## 2. Methods

### 2.1 Study registration

This systematic review has been registered on the International Prospective Register of Systematic Reviews (PROSPERO) under the registration code CRD42021281488. The review’s development will be based on the guidelines of the Preferred Reporting Items for Systematic Reviews and Meta-Analyses (PRISMA) (S1 File) [13].

### 2.2 Type of study and research participants

This review will include population-based studies that assess the environmental footprints of food consumption by adults and elderly worldwide.

### 2.3 Research question

What are the environmental footprints of diets assessed in population-based surveys conducted around the world?

### 2.4 Inclusion and exclusion criteria

We will include to this review population-based studies quantifying environmental footprints associated with the dietary intake of individuals. We will exclude articles resulting from systematic reviews, narrative reviews and meta-analysis, with future projections, scenarios, theoretical diets, studies with adolescents, children and pregnant women, as well as grey literature.

### 2.5 Search strategy

The evidence to be included in this research will be gathered based on search strategies previously defined. Initially, some strategies will be tested in the following electronic databases: Food Science and Technology Abstracts (FSTA), PubMed/Medline, Latin American and Caribbean Health Sciences Literature (LILACS) and ScienceDirect.

Search strategies will be developed based on keywords indexed in the Medical Subject Headings (MeSH) and Health Sciences Descriptors (*DeCS*). Boolean operators ’AND’ and ’OR’ will be combined with descriptors related to the Interest and Context of the ‘PICo’ defined for the research, contemplating sustainable diets and measurement methods such as indexes and scores.

Some examples of descriptors related to the Interest and Context are: environmental footprint, carbon footprint, water footprint, ecological footprint, diet, food consumption, dietary intake, food intake, and eating.

This is an example of a search strategy which may be used: (“carbon footprint*” OR “water footprint*” OR “ecological footprint*” OR “environmental footprint*”) AND (“diet*” OR “food consumption” OR “dietary intake” OR “food intake” OR “eating”).

### 2.6 Study selection process

**Fig 1:**
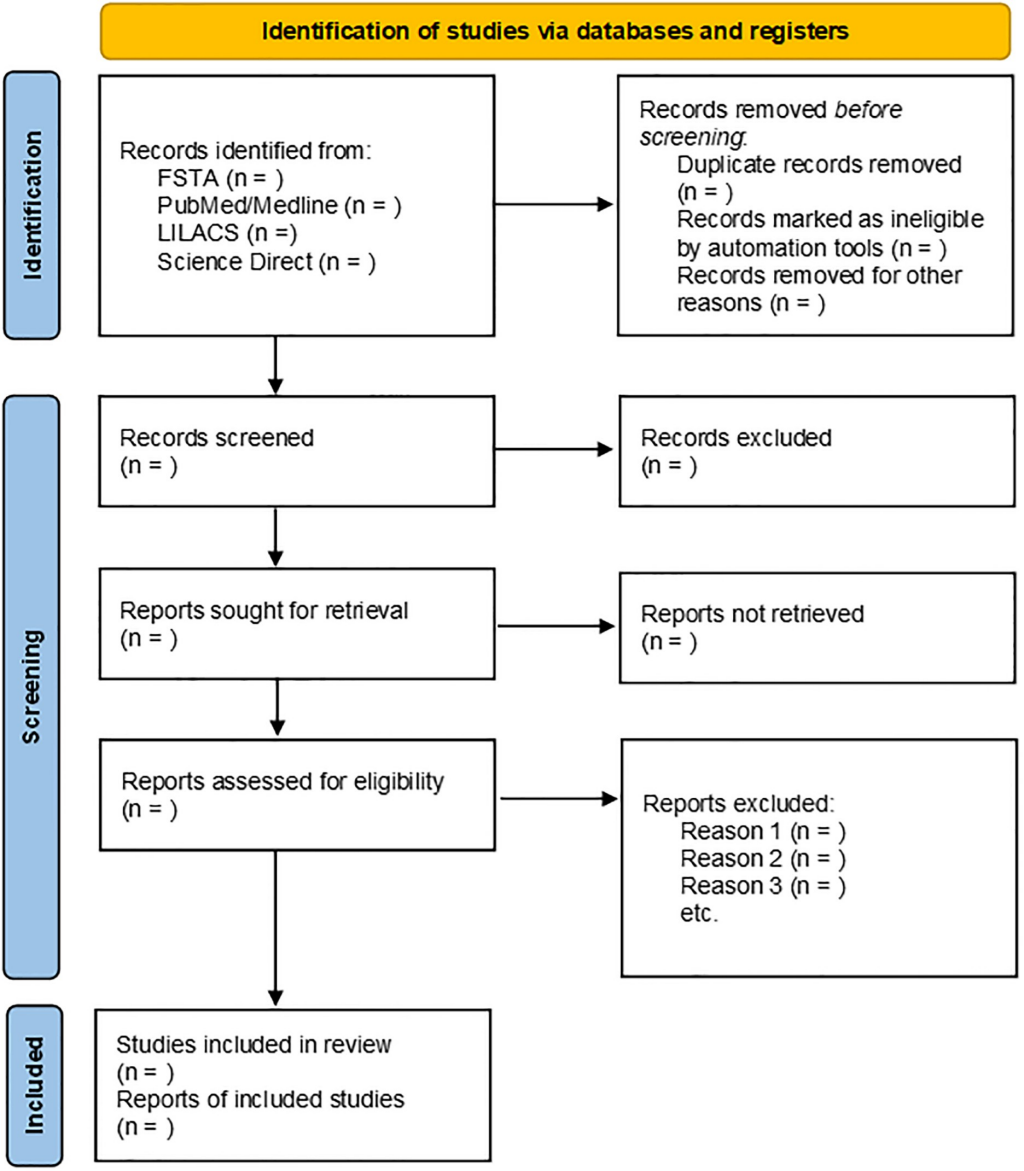
Flowchart of the study selection process.

Retrieved articles will be imported into *Rayyan Inteligent Systematic Review*^®^ for duplicate removal, initial screening of titles and abstracts, and selection after full reading. The search and selection of studies will be conducted independently by two reviewers, considering the eligibility criteria previously established, and in case there are conflicting decisions, a third reviewer will be consulted. The evidence selection process will be done as shown in the flowchart formulated based on the PRISMA recommendations [13] (Fig 1).

Still in this stage, the reviewers will independently perform the reading and evaluation of the full text of the potentially eligible studies retrieved. Any conflicting decisions between them on the eligibility of specific studies will be resolved through discussion with a third reviewer.

### 2.7 Data extraction and management

A pre-defined and previously tested (through a pilot sample) Microsoft Excel spreadsheet will be used to extract data from the included studies, for evaluation of study quality and evidence synthesis. The information extracted will include: author, year of publication, study design; population studied; study site; general and demographic characteristics of participants; diagnostic criteria used; study methodology; main results and mechanisms for risk of bias assessment.

### 2.8 Lost data

When extractions of results are unclear or incomplete, study authors will be contacted in order to obtain missing data. Contact will be made with the first or corresponding authors or co-authors by phone, email or mail. If we do not receive the necessary information, the data will be excluded from our analysis and will be addressed in the discussion section.

### 2.9 Methodological quality assessment

To assess the quality of the studies included in the research, two independent reviewers will use the New Castle Ottawa scale (cohort/case-control studies) and the AHRQ checklist for cross-sectional studies [14, 15]. A third reviewer will be consulted in case there are conflicting assessments.

Both scales aforementioned assess the following quality parameters: (i) selection of study groups (4 points), (ii) comparability of groups (2 points), (iii) verification of exposure or outcome and outcome for case-control and cohort studies, respectively (3 points).

### 2.10 Data synthesis

Initially, a descriptive synthesis of the content of the included studies will be carried out, such as population characteristics, study context, type of environmental footprint investigated in the study, footprint data obtained, methods used and results’ significance. In this stage, ‘Synthesis Without Meta-analysis (SWiM) in systematic reviews [16] reporting guideline will be used. There will be no minimum number of studies to be included in this review. The evidence synthesis will be ensured and the risk of bias due to selective publication will be controlled by following the steps previously described for critical appraisal of the studies and quality of evidence evaluation.

## 3. Discussion

The assessment of the sustainability of diets has gained more importance in establishing public policies, thus, clearly and comprehensively measuring the sustainability of food in the contexts of different populations and territories with different characteristics is necessary for the construction of healthier and more sustainable eating and environmental policies [17], which highlights the need to recognize and develop indexes and methodological approaches that characterize diets and their degrees of sustainability (more sustainable to less sustainable) [11].

In an initial analysis, there is a diversity of methodologies used to assess the environmental impact of population diets, from unidimensional [18, 19] to multidimensional [20] indicators. There are studies that assess population cuts of specific groups [21] to populational studies that estimate the environmental footprints of populations representative of countries [22, 23].

It is also important to know the methodology used, the type of study, the data source and its limitations. Certain dietary assessment methods may underestimate or overestimate individuals’ actual food consumption. One example is the food consumption assessment using FAOSTAT data on food availability per capita at a national level. Although this source is frequently used to assess individual food consumption, this method may not represent actual consumption [9, 24]. Thus, while some studies assess the environmental impact of food supply [25, 26] and/or food availability [18, 27], our review proposes to discuss studies which assess habitual food consumption and/or dietary recall.

The same may be observed regarding environmental footprints. Depending on the source used to calculate footprints values, this estimate may not adequately reflect the impact of a population’s food consumption, since certain sources represent the reality of a specific country or are based on global averages. The difference between agricultural and livestock systems, for example, impacts the final values of GHG emissions, water and land use, making it relevant to consider where the food was produced and consumed to bring estimates closer to reality [9, 28].

Furthermore, the clear definition of system boundaries when considering the life-cycle assessment methodology is essential as it impacts the accuracy of the analysis. Assessments which consider the entire life cycle of products are called “Cradle-to-grave” assessments, which however, are not used in many studies, excluding stages such as transport, storage, consumption and waste management. Excluding these steps may impact estimated footprint values, in some cases underestimating them [8, 29].

Providing information on the food consumption of populations based on sustainability indicators will, for example, enable consumers to make more conscious choices, favoring market changes driven by demand for products that support sustainable food systems [6], besides strengthening the development of public policies focused on promoting educational actions, healthier and more sustainable environments and food systems.

In this sense, estimating the environmental footprints of food consumption by populations seems to be a viable and essential strategy, since it will provide an overview of the environmental impact around the world, considering the diversity of territories and allowing discussions on dietary patterns and the characteristics of food systems in each country.

Previous systematic reviews have sought to investigate the relationship between environmental footprints and sustainable diets. These studies, however, generally measure the methods used [17] or directly assess only one type of environmental footprint [9], in addition to including studies that evaluated not only food consumption, but also food availability and future projections [9, 10, 17].

As limitations in the development of the proposed systematic review, we can cite: the inclusion of studies that will not generate high-quality evidence, the absence of studies that evaluate individual food consumption, as it was verified in an initial search the use of methodologies that use estimates of food consumption based on the purchase, supply or food availability at home. In addition, the possibility of heterogeneity between studies may bring difficulties to perform the meta-analysis.

## 4. Conclusion

Our study will expand the possibilities of the scientific community to identify the main environmental footprints used to assess the environmental impact of food consumption by populations, as well as the instruments and methodologies used in this assessment. Thus, it is expected to fill a gap, since, as far as we know, there are no studies that propose similar analyses. Thus, the systematic review produced from this protocol will synthesize the main evidence, allowing us to assess the health and environmental impacts of current diets, as well as establish parameters for the assessment of the environmental footprint in future research for the development of public policies to promote healthy diets and sustainable.

## Supporting information

S1 File PRISMA—P 2015 checklist. (DOCX)
